# Mechanisms of Dexmedetomidine in Neuropathic Pain

**DOI:** 10.3389/fnins.2020.00330

**Published:** 2020-05-05

**Authors:** Yang Zhao, Jianshuai He, Ning Yu, Changxin Jia, Shilei Wang

**Affiliations:** Department of Anesthesiology, The Affiliated Hospital of Qingdao University, Qingdao, China

**Keywords:** neuropathic pain, dexmedetomidine, adrenergic receptor, neuroprotective effect, α2 adrenergic receptor

## Abstract

Dexmedetomidin is a new-generation, highly selective α2 adrenergic receptor agonist with a large number of advantages, including its sedative and analgesic properties, its ability to inhibit sympathetic nerves, its reduced anesthetic dosage, its hemodynamic stability, its mild respiratory depression abilities, and its ability to improve postoperative recognition. Its safety and effectiveness, as well as its ability to provide a certain degree of comfort to patients, make it a useful anesthetic adjuvant for a wide range of clinical applications. For example, dexmedetomidine is commonly used in patients undergoing general anesthesia, and it also exerts sedative effects during tracheal intubation or mechanical ventilation in intensive care unit patients. In recent years, with the deepening of clinical research on dexmedetomidine, the drug is still applied in the treatment of spastic pain, myofascial pain, neuropathic pain, complex pain syndrome, and chronic headache, as well as for multimodal analgesia. However, we must note that the appropriateness of patient and dose selection should be given attention when using this drug; furthermore, patients should be observed for adverse reactions such as hypotension and bradycardia. Therefore, the safety and effectiveness of this drug for long-term use remain to be studied. In addition, basic experimental studies have also found that dexmedetomidine can protect important organs, such as the brain, heart, kidney, liver, and lung, through various mechanisms, such as antisympathetic effects, the inhibition of apoptosis and oxidative stress, and a reduction in the inflammatory response. Moreover, the neuroprotective properties of dexmedetomidine have received the most attention from scholars. Hence, in this review, we mainly focus on the characteristics and clinical applications of dexmedetomidine, especially the role of dexmedetomidine in the nervous system and the use of dexmedetomidine in the relief of neuropathic pain.

## Dexmedetomidine

### Discovery and Clinical Application of Dexmedetomidine

Dexmedetomidine is a dextrorotatory isomer of the racemic mixture medetomidine (Keating, 2015). Hoy et al. investigated whether the activation of α2-adrenergic receptors could change the amount of inhaled anesthetic, found that the α2-adrenergic receptor agonist dexmedetomidine itself was an anesthetic with a calming and hypnotic effect (Hoy and Keating, 2011). Then, they immediately recruited relevant patents and further launched a series of studies, and the relevant research results were also utilized to the human body, which led to the emergence of dexmedetomidine as a sedative (Barends et al., 2017). It was subsequently developed in 1990 by OrionPharma (Finland) and Abbott Labs (**United States**), and afterward, it was used in intensive care units (ICUs) as a short-term sedative (<24 h) approved by the United States government in 1999 (Keating, 2015). In 2008, dexmedetomidine was approved for non-tracheal intubation patients and perioperative treatment (Cruickshank et al., 2016). However, in recent years, accumulating evidence has demonstrated that, clinically, dexmedetomidine has an analgesic effect on ischemic pain, acute postoperative pain, refractory cancer pain, and herpetic stomatitis after living lung transplantation (Hoy and Keating, 2011). In addition, a large number of studies in different animal models have also confirmed that dexmedetomidine has analgesic and anti-inflammatory effects in postoperative, streptozotocin-induced, Freund’s complete adjuvant, formalin, and carrageenan-induced inflammation as well as acute nociceptive and neuropathic pain (Mahmoud and Mason, 2015; Sottas and Anderson, 2017).

### Basic Pharmacology of Dexmedetomidine

#### Structure of Dexmedetomidine

Dexmedetomidine is a newly developed α2 adrenergic receptor agonist, an imidazole derivative with the chemical name 4-[(1R)-1-(2,3-dimethylphenyl)ethyl]-3H-imidazole hydrochloride (Greenberg et al., 2017). Dexmedetomidine is a highly potent and highly selective α2 adrenergic receptor agonist with a receptor selectivity (α2/α1) of 1,620:1, but clonidine receptor selectivity (α2/α1) is 220:1 (Giovannitti et al., 2015). Moreover, the intrinsic activity of dexmedetomidine is stronger than that of clonidine (3:1) (Pichot et al., 2012). The elimination half-life of dexmedetomidine is approximately 2 h, while the elimination half-life of clonidine is 8.6 ± 1.5 h. Thus, due to its unique receptor activity and short half-life, as well as its special pharmacological effects, dexmedetomidine has broad application prospects in clinical anesthesia (Mavropoulos et al., 2014; Greenberg et al., 2017).

#### Specificity of the Dexmedetomidine Effect: Acceptor-Mediated Entrance Into the Neuronal Cytosol

Dexmedetomidine is a G-protein-coupled α2 receptor agonist (Rodriguez-Gonzalez et al., 2016). The α2 adrenergic receptors in humans mainly contain α2A, α2B, and α2C and are widely distributed in the central nervous system (CNS), peripheral nervous system (PNS), autonomic ganglia, and other organ tissues, including the blood vessels, liver, kidney, pancreas, and platelets (Baron, 2009). Different subtypes of α2 adrenergic receptors have different functions. The α2A receptor is considered to be the major presynaptic inhibitory feedback receptor, controlling the exocytosis of adrenergic neurons (Scholz et al., 2019). Thus, deletion of a gene encoding the α2A receptor results in elevated blood pressure, increased heart rate, and easy progression to cardiac hypertrophy and heart failure. In addition, the α2A receptor or the α2 receptor agonist is required for sedation, analgesia, epilepsy regulation, and platelet aggregation (Baron, 2009; Scholz et al., 2019). The α2B receptor is mainly distributed in peripheral vascular smooth muscle and can cause a transient hypertensive response after activation (Jiang et al., 2017). The α2B receptor in the spinal cord is a basic component of the regulation of nitrogen monoxide analgesia by descending noradrenergic neurons (Jiang et al., 2017; Mantz et al., 2011). The α2C receptor is mainly distributed in the hippocampus, basal ganglia, olfactory bulb system, and cerebral cortex and is involved in the regulation of a variety of complex memory and behavioral functions (Mantz et al., 2011). Dexmedetomidine is a highly selective activator of the α2A receptor, acting on the nucleolus of the nucleus, and it can have a sedative and hypnotic effect; its action on the spinal cord can produce analgesic effects, while its action on the peripheral and CNSs can play a role in inhibiting sympathetic excitation (Kenney et al., 2014; Giovannitti et al., 2015).

#### Inhibition of Neurotransmitter Release

In the CNS, the most important method of synaptic transmission is neurochemical transmission (Pocock and Kettenmann, 2007). The neurotransmitter, in synaptic transmission, is a specific chemical that acts as a “messenger” (Lee, 2013). It is bound to the corresponding postsynaptic membrane receptor immediately after release from the presynaptic membrane, producing synaptic depolarization potentials or superpolar potentials, resulting in increased or decreased excitability of the postsynaptic nerve (Zindler and Zipp, 2010; Lee, 2013). Thus, the release of neurotransmitters is directly related to the production of neural excitability. Previous studies have shown that the release of neurotransmitters, such as catecholamine and norepinephrine, is closely associated with hypoxic brain damage (Zindler and Zipp, 2010). Catecholamine increases neuronal sensitivity to glutamate during neuronal ischemia, while norepinephrine metabolites promote oxidative stress, which further exacerbates damage to nerve tissue (Starke, 2014). Furthermore, it was discovered that dexmedetomidine could reduce the release of catecholamines in nerve endings by reducing the activity of the sympathetic nervous system (Wu et al., 2020). It was also found to directly act on α2 receptors in monoaminergic neurons and dendrites in the brain to inhibit the secretion of catecholamines (Obata, 2017). Hence, the above study concluded that dexmedetomidine could reduce neuronal damage by inhibiting neurotransmitter release, which improved ischemic perfusion and metabolic disorders (Wang et al., 2019).

#### Modulation of Voltage-Gated Calcium Channels and Glutamate Excitotoxicity

Excessive release of glutamate may induce an increase in intracellular Ca^2+^ expression levels, resulting in increased production of oxygen free radicals, thereby interfering with mitochondrial function and activating proteases, ultimately leading to neuronal death (Crupi et al., 2019). Wang et al. (2019) found that dexmedetomidine could upregulate the expression of excitatory amino acid transporter 1 by increasing the release of the *N*-methyl-D-aspartate receptor and then that it could trigger a decrease in glutamate release to alleviate nerve cell damage in neonatal rats caused by isoflurane. Therefore, this study implies that dexmedetomidine might exert a neuroprotective effect by inhibiting the influx of calcium ions and promoting the release of glutamate. Furthermore, another study reported that dexmedetomidine could enhance the expression of excitatory amino acid transporter 3 (EAAT3) in glutamate in *Xenopus* oocytes in a concentration-dependent manner, suggesting that dexmedetomidine could decrease the concentration of extracellular glutamate by increasing the expression of EAAT3 and could exert neuroprotective and anticonvulsant effects (Do et al., 2014). These effects were mediated by protein kinase C and phosphatidylinositol-3-hydroxykinase. Additionally, KM Chiu et al. (2011) observed that dexmedetomidine activated α2A adrenergic receptors in cerebral cortical nerve endings, inhibited glutamate release, and further reduced extracellular glutamate deposition by suppressing the activity of N-type and P/Q-type voltage-gated calcium channels and mitogen-activated protein kinases (MAPKs), which ultimately produced neuroprotective effects.

## Neuropathic Pain

Neuropathic pain, a type of chronic and potentially disabling pain caused by disease or injury to the somatosensory nervous system and spinal cord, can be accompanied by various chronic conditions, such as viral infections (e.g., postherpetic neuralgia), autoimmune diseases, cancers, and metabolic disorders (e.g., diabetes mellitus); neuropathic pain is one of the most intense types of chronic pain, resulting in a major economic burden and a serious public health issue, with an estimated prevalence of 7–10% adults worldwide (Cohen and Mao, 2014; Knezevic et al., 2017). According to the underlying etiology and the anatomical location of the specific lesion, clinical neuropathic pain is divided into four main types, namely, painful peripheral neuropathies, central pain syndromes, complex painful neuropathic disorders, and mixed-pain syndromes (Baron, 2009; Binder and Baron, 2016). Painful peripheral neuropathies are caused by ischemic, traumatic, inflammatory, infectious, metabolic, or compressive damage to the PNS, such as polyneuritis, phantom limb pain, diabetic peripheral neuralgia, and postherpetic neuralgia (Stacey, 2005; Brouwer et al., 2014; Scholz et al., 2019). Central pain syndromes are characterized by primary pain resulting from dysfunction or damage to the CNS pain conduction pathway, mainly involving cerebrovascular disease, spinal cord trauma, etc. (Bettini and Moore, 2016; Scholz et al., 2019). Complex painful neuropathic disorders are also called “complex regional pain syndromes (CRPS),” which may develop as a disproportionate consequence of trauma typically affecting the limbs (Colloca et al., 2017). Mixed pain syndromes are characterized by a combination of nociceptive and neuropathic pain (Baron, 2009). Currently, the incidence of neuropathic pain is high, and treatment is difficult, which seriously affects the work and quality of life of patients (Vranken, 2012; Yan et al., 2017). Nevertheless, the pathogenesis of neuropathic pain is complex and has not yet been clarified.

## Neuroprotective Effects and Mechanisms of Dexmedetomidine

### Neuroprotective Effect of Dexmedetomidine

In recent years, dexmedetomidine has received extensive attention from scholars due to its good sedative effect (Keating, 2015). Moreover, it has been widely used as an anesthesia adjuvant for clinical anesthesia and severe disease (Baron, 2009). Additionally, growing evidence has also shown that dexmedetomidine can produce neuroprotective effects through various mechanisms, such as antioxidation and anti-infection activities, the inhibition of apoptosis, the promotion of neurogenesis, and the influence of cell signaling pathways (Shen et al., 2017; Wu et al., 2018). The neuroprotective effect of dexmedetomidine was first proposed by Hoffman et al. (1993). It was found that, in a rat model of cerebral ischemia, intraperitoneal injection of dexmedetomidine before cerebral ischemia significantly reduced plasma catecholamine levels, thereby reducing cerebral ischemic damage (Rodriguez-Gonzalez et al., 2016; Wang S.L. et al., 2017). Subsequently, the neuroprotective effect of dexmedetomidine was further examined by a large number of researchers (Rodriguez-Gonzalez et al., 2016; Jiang et al., 2017). For example, Sato et al. (2010) uncovered that the neurological function of ischemic injury was significantly improved in rats with focal cerebral ischemia after dexmedetomidine, the survival of neurons in the CA1 region of the ischemic hippocampus increased, and dexmedetomidine combined with hypothermia could enhance the above effects. Zhu et al. (2013) revealed that dexmedetomidine was applied in a rat model of cerebral ischemia–reperfusion injury, which significantly reduced neurological deficits, cerebral infarction volume, cerebral edema, and neuronal death in the hippocampal CA1 and cerebral cortex. Liao et al. (2014) discovered that dexmedetomidine could decrease isoflurane-induced neuroapoptosis by activating the phosphatidylinositol 3-kinase (PI3K)/protein kinase B (Akt) pathway and suppressing the p38/ **MAPK** pathway in the hippocampus of neonatal rats. Furthermore, the role of dexmedetomidine in neuropathic pain has also gradually been disclosed (Di Cesare Mannelli et al., 2017). For instance, intrathecal injection of dexmedetomidine relieved neuropathic pain through the modulation of MPK3/extracellular regulated protein kinase (ERK)1/2 in a mouse model of neuropathic pain (Qian et al., 2019), dexmedetomidine ameliorated neuropathic pain by hindering spinal P2**X**7R expression and ERK phosphorylation in an animal model of chronic constriction injury (Lin et al., 2018), and dexmedetomidine attenuated neuropathic hypersensitivity and inflammation partially by restraining ***N***-methyl-D-aspartate receptor 2B (NR2B), nuclear factor-kappa B (NF-κB), and inducible nitric oxide synthase (iNOS) expression in the spinal cord of rats with neuropathic pain resulting from chronic constriction injury of the sciatic nerve (Liang et al., 2017). Therefore, the neuroprotective effect of dexmedetomidine is unquestionable, but its specific neuroprotective mechanism has not yet been fully elucidated.

### Mechanism of Dexmedetomidine’s Protective Effects on the Nervous System

#### Anti-inflammatory Influences of Dexmedetomidine

Brain injury can cause the release of a variety of inflammatory factors, and the inflammatory response is an important pathogenesis of brain injury (Jassam et al., 2017; Magalhaes et al., 2019). The neuroprotective effect of dexmedetomidine is also related to its inhibition of the inflammatory response (Zhang et al., 2015). The immune response plays an important role in neuroprotection (Li et al., 2017). Dillon et al. (2017) found that dexmedetomidine could reduce the levels of inflammatory cytokines, such as tumor necrosis factor-α (TNF-α) and interleukin-1 (IL-1), by activating the α2-adrenergic receptor signaling pathway. Studies have shown that dexmedetomidine can attenuate the inflammatory responses associated with ischemia/reperfusion injury by inhibiting NF-κB activation (Wang L. et al., 2017). In addition, Kim et al. (2017) discovered that dexmedetomidine could induce an anti-inflammatory response in cerebral ischemia–reperfusion injury through inactivation of the Toll-like receptor 4 (TLR4)/NF-κB pathway, which is an important mechanism of neuroprotection.

#### Anti-excitotoxicity Actions of Dexmedetomidine

Cerebral ischemia and hypoxia can lead to increased release of glutamate and aspartate (Hsieh et al., 2017). If the concentration of glutamic acid and aspartic acid in the extracellular fluid is increasing, an excessive concentration of the excitatory amino acid neurotransmitter glutamate causes *N*-methyl-D-aspartate receptor overactivation, which finally leads to neuronal cell apoptosis (Zhang et al., 2017). However, in the case of cerebral ischemia and hypoxia, dexmedetomidine could reduce the release of glutamate by acting on cell membrane ion channels and, on the other hand, could increase the production of brain-derived growth factor (BDNF) in astrocytes to protect against glutamate neurotoxicity (Liu et al., 2017). In addition, it was found that dexmedetomidine could not only reduce glutamate content after cerebral ischemia but also increase gamma aminobutyric acid content and decrease brain ultrastructural damage, indicating that dexmedetomidine might exert its neuroprotective effects by downregulating excitatory neurotransmitters and therefore upregulating inhibitory neurotransmitters (Alam et al., 2017; Endesfelder et al., 2017).

#### Inhibition of Neuronal Apoptosis by Dexmedetomidine

Neuronal necrosis often occurs early in ischemia and hypoxia damage, while neuronal apoptosis can continue to occur within a few weeks after ischemia (Sun et al., 2015; Li C. et al., 2018). Apoptosis is defined as programmed cell death mediated by many genes, among which the important apoptosis regulatory proteins are Bcl-2, BCL-XL, and Bax (Wang et al., 2020). Experimental studies have revealed that dexmedetomidine can increase BDNF expression by directly acting on the α2A receptor and then modulating the ERK1/2 pathway, and BDNF can in turn promote the expression of Bcl-2 and BCL-XL by activating the PI3K/Akt signaling pathway and MAPK pathway and thus suppressing the p38 and c-Jun N-terminal kinase (JNK) pathways, which eventually leads to a decrease in neuronal apoptosis (Bell et al., 2014; Li Z.C. et al., 2018). Moreover, Kuang et al. (2015) revealed that dexmedetomidine could effectively alleviate ketamine-induced neuronal apoptosis in the hippocampal CA1 region as well as learning and memory dysfunction in rat brain development, and dexmedetomidine alone had no obvious neurotoxicity in 7-day-old neonatal rats.

#### Roles of Dexmedetomidine in Astroglia

Glial cells are supportive cells widely distributed in the nervous system (Watzlawik et al., 2010). They support and guide neuronal migration, participate in the repair and regeneration of the nervous system or immune responses, form myelin and blood–brain barriers, and maintain potassium ion concentrations outside nerve cells (Watzlawik et al., 2010; Machado et al., 2016). Glial cells include astrocytes, oligodendrocytes, and microglia in the CNS (von Bernhardi et al., 2016). In the CNS, astrocytes connect capillaries and neurons through perivascular limbs, which can transport nutrients and eliminate metabolites to neurons (Zuchero and Barres, 2015). It has been found that dexmedetomidine can inhibit the astrocyte-induced overactivation caused by ischemia–reperfusion and can reduce the expression of glial fibrillary acidic protein and inflammatory factor TNF-α, which suggests that dexmedetomidine has neuroprotective effects on cerebral ischemia–reperfusion injury in rats (Freeman et al., 2015; Shi et al., 2018).

#### Antioxidative Effects of Dexmedetomidine

The brain is the most oxygen consuming of all organs in the human body but lacks antagonists against oxidative stress in CNS neurons, so neuronal cells are easily damaged by the release of free radicals (Salim, 2017; Dumitrescu et al., 2018). Cerebral ischemia and hypoxia damage can result in an increase in reactive oxygen species in the brain tissue, which in turn causes oxidative damage to cellular macromolecules such as nucleic acids and proteins to induce cell death (Torres-Cuevas et al., 2019; Zhou et al., 2019). Studies have found that inhalation of high-concentration oxygen in rat pups produces inflammatory brain damage induced by oxidative stress, which is characterized by increased apoptosis, a decreased ratio of glutathione/oxidized glutathione disulfide, increased lipid peroxidation, and increased IL-1β levels (Zhenyukh et al., 2017). Nevertheless, dexmedetomidine treatment has been observed to alleviate or eliminate these deleterious effects induced by these oxidation reactions (Fu et al., 2017; Xu et al., 2019).

#### Dexmedetomidine Regulates Synaptic Plasticity and Hippocampal Long-Term Potentiation

Synaptic plasticity, including structural and functional plasticity, provides an important basis for learning and memory (Singh and Abraham, 2017). However, hippocampal long-term potentiation refers to the long-term enhancement of synaptic transmission efficiency produced by the tonic stimulation of neural pathways, which can reflect the physiological activities of neurons during the formation of learning and memory and is a manifestation of synaptic plasticity (MacDonald et al., 2006). Previous research reported that 7-day-old rats were given moderate doses of dexmedetomidine, causing mild respiratory depression, but these doses did not impair synaptic plasticity in adulthood, which implied that dexmedetomidine might be relatively safe during development (Tachibana et al., 2012). Additionally, cerebral ischemia and hypoxia could cause long-term potentiation after ischemia (i-LTP), while the combined application of dexmedetomidine could not only restrain the release of noradrenaline and glutamate from the presynaptic membrane, which triggered presynaptic inhibition and weakened i-LTP, but also hyperpolarize the postsynaptic membrane by reducing the activation of the *N*-methyl-D-aspartate receptor and hindering the β-receptor and its downstream cyclic AMP (cAMP)/protein kinase A (PKA) pathway; thus, β-cAMP/PKA intracellular signaling pathways might be involved in the regulation of improving synaptic plasticity in response to dexmedetomidine (Niittykoski et al., 2000; Takamatsu et al., 2008; Kato et al., 2014).

#### Suppression of Sympathetic Activity and Regulation of Cerebral Blood Flow

Studies have shown that dexmedetomidine reduces cerebral blood flow and the brain metabolic rate in healthy volunteers (Wang et al., 2013). In patients who undergo surgery, dexmedetomidine can lessen intracranial pressure, reduce cerebral oxygen metabolism, maintain the balance of cerebral oxygen supply and demand, and diminish agitation and postoperative sputum (Jiang et al., 2017). Cheng et al. also confirmed that in cerebral ischemia–reperfusion, dexmedetomidine decreased the area of cerebral infarction in the early stage of ischemia–reperfusion by reducing the heterogeneity of mixed venous oxygen saturation, reducing regional cerebral blood flow and cerebral oxygen consumption, and maintaining cerebral oxygen supply and demand (Cheng et al., 2018). In addition, it has been reported that dexmedetomidine is dependent on the α2 adrenergic receptor and preferentially bound to the inactivated sodium channel and acetylcholine receptor channel in the supraspinatus ganglion in rats and that it then inhibits sodium channels and acetylcholine receptor channels in a dose-dependent manner (Yang et al., 2015). Moreover, it has been further demonstrated that the inhibition of dexmedetomidine in sodium channels and acetylcholine receptor channels cannot be blocked by yohimbine, phentolamine, or atropine, which might be suggestive of the mechanism of the central antisympathetic activity of dexmedetomidine (Yang et al., 2015).

#### Induction of the Low-Temperature Effect of Dexmedetomidine

McAdams et al. (2015) administered dexmedetomidine or combined hypothermia treatment to neonatal rats and subsequently discovered that acute or persistent exposure to dexmedetomidine did not cause abnormal pathological changes in the brain tissue of newborn rats, and there was a correlation between plasma dexmedetomidine concentration and brain tissue dexmedetomidine concentration. Furthermore, it was also found that the dexmedetomidine level in rat brain tissue reached a peak within 15 min of administration in the low-temperature group, and the central temperature was reduced from 32 to 30°C within 30 min after administration, indicating that dexmedetomidine combined with hypothermia might be used to treat acute craniocerebral injury (Lewis et al., 2015).

## An Overview of the Clinical Evidence of Dexmedetomidine Analgesic Efficacy

### Molecular Mechanism of Analgesic Efficacy of Dexmedetomidine

There are four different types of α2 adrenergic receptor subtypes in the human body, namely, α2A, α2B, α2C, and α2D receptors (Giovannitti et al., 2015). Current studies suggest that the α2A receptor mediates the anesthetic and analgesic effects of dexmedetomidine (Mantz et al., 2011). The mechanism of dexmedetomidine analgesia has not been fully clarified, but the main mechanisms might be as follows: (1) peripheral analgesic effect, an analgesic effect produced by inhibiting the transmission of pain signals by inhibiting Aδ and C fibers; (2) central analgesic effect, mainly depolarizes the blue plaque and the descending noradrenergic pathway of the spinal cord to the presynaptic membrane, inhibiting the release of substance P and other nociceptive peptides in the presynaptic membrane and thereby inhibiting the spinal cord via the transmission of angular noxious stimuli, which in turn terminates the signaling of pain; and (3) local analgesic effect, modulation of hyperalgesia by stimulating the α2 receptor (Zhang and Bai, 2014). In the past, opioids were mainly used to relieve acute postoperative pain, but opioids can cause toxic side effects such as nausea, vomiting, respiratory depression, and itchy skin (Gupta and Bah, 2016). However, the highly selective α2 receptor agonist dexmedetomidine has good sedative and analgesic effects, has little effect on the circulatory system, and does not inhibit breathing (Giovannitti et al., 2015). It is now being used as an auxiliary drug due to its good anesthesia and analgesia (Cohen and Mao, 2014). In terms of postoperative multimodal analgesia, dexmedetomidine reduces the amount of opioids used, and patient comfort is high (Hoy and Keating, 2011; Cohen and Mao, 2014). Although the current literature reports that dexmedetomidine only plays a supporting role in analgesia, the mode of administration of dexmedetomidine plus other analgesics has brought new ideas and compatibility to postoperative analgesia and promotes the improvement of pain treatment (Vranken, 2012). The advantages of dexmedetomidine for postoperative analgesia primarily include the following: (1) the diversity of analgesic methods, dexmedetomidine can be administered not only by intravenous or intrathecal administration but also by topical medication, which can enhance the postoperative analgesic effect; (2) it is the only sedative that has an analgesic effect and has a wide range of safety, dexmedetomidine not only enhances postoperative analgesia but also prevents postoperative sedation; and (3) the wide range of applications and organ protection, dexmedetomidine can be used for postoperative analgesia in various operations. It can be combined with analgesics with different mechanisms of action to exert its superior effects, such as reducing the incidence of postoperative agitation and spasm (Pichot et al., 2012). However, in addition to the above advantages, dexmedetomidine has side effects in its range of conventional use. Among them, the most common side effects are bradycardia and hypotension (Weerink et al., 2017). Therefore, the dosage and the mode of administration should be strictly controlled. If a high concentration of the load is given, hypotension, high blood pressure, or bradycardia can result.

At present, in view of the better analgesic effect of dexmedetomidine, in addition to being used for surgical analgesia, the drug can also be used for labor analgesia, gastrointestinal endoscopy analgesia, neuropathic pain, cancer pain, and so on (Hoy and Keating, 2011; Vranken, 2012). Moreover, a growing number of studies have confirmed that dexmedetomidine has an analgesic effect on neuropathic pain, and its mechanism may be related to the downregulation of the microglia P2X4 receptor, phosphorylated p38 protein kinase, and brain-derived neurotrophic factor (Zhou et al., 2014). The detailed studies of dexmedetomidine in relation to neuropathic pain are listed in Table 1.

**TABLE 1:** Studies of dexmedetomidine for neuropathic pain.

**Model**	**Results**	**References**
A mouse model induced by chronic constriction injury	Dexmedetomidine play an analgesic role in neuropathic pain through the regulation of MKP3/ERK1/2 signaling pathway.	Qian et al., 2019
A randomized prospective double-blinded trial	Dexmedetomidine is considered as an adjuvant to bupivacaine paravertebral block and further reliver pain during the early postoperative hours, and it provided a better effect on chronic postoperative pain.	Abd-Elshafy et al., 2019
Rats diabetic neuropathy pain model	Dexmedetomidine alleviated diabetic neuropathy pain in rats via hindering inflammation and astrocyte activation, which may be attributed to downregulation of the Wnt 10a/β-catenin signaling pathway.	Zhong et al., 2018
A rat model induced by chronic constriction injury	The intrathecal injection of dexmedetomidine may improve the behavioral ability of rats with chronic neuralgia and reduce the degree of pain, which may be associated with the inhibition of the expression of PKC in the spinal dorsal horn.	Li and Zhang, 2018
A rat model induced by chronic constriction injury	Dexmedetomidine changed the expressions of NR2B and GABAA-α1 following peripheral nerve injury in rats to manage neuropathic pain.	Chen et al., 2018
A rat model induced by chronic constriction injury	Dexmedetomidine relieves neuropathic pain by inhibiting hyperpolarization-activated cyclic nucleotide-gated currents in dorsal root ganglia neurons.	Yang et al., 2018
A rat model of skin/muscle incision and retraction	Peripheral administration of dexmedetomidine improves mechanical and heat hyperalgesia and mitigates postoperative pain.	Huang et al., 2017
A rat model of neuropathic pain	The therapeutic effectiveness of dexmedetomidine in neuropathic pain may be through inhibition of proinflammatory cytokines, primarily IL-6 and TNF-α.	Farghaly et al., 2016
Rats with nerve-ligation injury	The antinociceptive of dexmedetomidine was confirmed in neuropathic pain treatment.	Farghaly et al., 2014
A patient with metastatic sarcoma suffering from an acute postoperative neuropathic pain crisis	Dexmedetomidine should be considered as a viable treatment alternative for acute postoperative neuropathic pain crisis.	O’Neil et al., 2014
Rats with spared nerve injury	Dexmedetomidine could attenuate the neuropathic pain via down-expressing P2X4Rs, p-p38, and BDNF in microglia of spinal dorsal horn.	Zhou et al., 2014
Rats subjected to chronic constriction injury	Dexmedetomidine played an anti-nociceptive effect via mediating IL-18 signaling pathway in microglia.	Chandrasekar et al., 2005
A rat spinal nerve ligation model	Intraplantar injection of dexmedetomidine produced an antiallodynic effect in spinal nerve ligation-induced neuropathic pain.	Lee et al., 2013
Vincristine-evoked painful neuropathic rats	Dexmedetomidine shows a dose-dependent antiallodynic effect on mechanical and cold stimuli in vincristine-evoked neuropathic rat models.	Park et al., 2012
A rat model of neuropathic pain induced by spinal nerve ligation	Dexmedetomidine decreases hyperalgesia in neuropathic pain by increasing acetylcholine in the spinal cord.	Kimura et al., 2012
Nerve injury-induced pain	Dexmedetomidine exerts antinociceptive effects in the development of neuropathic pain after peripheral nerve injury.	Ulger et al., 2009
The spinal nerve ligation model of neuropathic pain in rats	Systemic administration with dexmedetomidine could prevent and treat the nerve injury-induced pain.	Ma et al., 2018

### Clinical Evidence of Dexmedetomidine Analgesic Efficacy

Moreover, there have been some randomized clinical trials on the use of dexmedetomidine for neuropathic pain, which are exhibited in Table 2. Andjelkovic et al. conducted a randomized controlled clinical trial to evaluate the analgesic efficacy of dexmedetomidine in operation. Thirteen carcinoma patients received infusion of dexmedetomidine (0.5 μg/kg/h) and stopped infusion at the end of surgery. It was found that dexmedetomidine would significantly increase piritramide consumption than lidocaine. Dexmedetomidine failed to improve bowel function but effectively reduce propofol consumption during surgery (Andjelkovic et al., 2018). Xu et al. (2017) investigated whether lidocaine combined with dexmedetomidine infusion was superior in controlling pain and recovery of bowel function in 240 women undergoing elective abdominal hysterectomy. In this study, dexmedetomidine + lidocaine reduced the time to first bowel sounds, visual analog scale (VAS) score, and postoperative fentanyl requirement. The combination application significantly improved postoperative pain and enhanced recovery of bowel function (Xu et al., 2017). Naik et al. (2016) enrolled 142 patients with more than three levels of thoracic and/or lumbar spine surgery receiving dexmedetomidine of 0.5 μg/kg/h in a randomized double-blind study. It was found that dexmedetomidine could increase the incidence of bradycardia and phenylephrine. Intraoperative dexmedetomidine does not reduce postoperative opioid consumption or improve pain scores after multilevel deformity correction spine surgery (Naik et al., 2016). Kido et al. (2014) reported a case of successful procedural sedation using dexmedetomidine in a 68-year-old woman undergoing left Gasserian ganglion block for intractable trigeminal neuralgia. Dexmedetomidine (DEX) was chosen to provide an effective sedation and clear communication about the injection of drugs or thermocoagulation. After 15 min of DEX administration at 0.8 μg/kg/h, nerve block needle insertion was bearable. The patient could feel a weakening of the sensation of her left maxilla and mandible after receiving a 0.4-ml mepivacaine test dose. DEX sedation for interventional pain management during procedures such as Gasserian ganglion block may be useful (Kido et al., 2014). These studies provide an important theoretical basis for the application of dexmedetomidine in neuropathic pain.

**TABLE 2:** Randomized clinical trials of dexmedetomidine for neuropathic pain.

**Pain condition**	**Number of participants and does/delivery of route**	**Primary outcomes**	**References**
Perioperative opioid consumption in laparoscopic intestine resection	59 participants, continuous intravenous infusion of dexmedetomidine (0.5 μg/kg/h)	Dexmedetomidine reduced intraoperative propofol consumption.	Andjelkovic et al., 2018
Postoperative pain and bowel function recovery after abdominal hysterectomy	240 women, Received dexmedetomidine infusion (0.5 μg/kg loading, 0.4 μg/kg/h infusion)	Dexmedetomidine infusion improved postoperative pain and enhanced recovery of bowel function undergoing abdominal hysterectomy.	Xu et al., 2017
Postoperative opioid consumption and pain after major spine surgery	142 participants, 1 μg/kg load followed by a continuous infusion of 0.5 μg/kg/h	Intraoperative dexmedetomidine does not reduce postoperative opioid consumption or improve pain scores after multilevel deformity correction spine surgery.	Naik et al., 2016
Gasserian ganglion block for trigeminal neuralgia	A case of successful procedural sedation using dexmedetomidine, 0.8 μg × kg^–1^ × h^–1^	Dexmedetomidine sedation for interventional pain management during procedures such as Gasserian ganglion block may be useful.	Kido et al., 2014
			

## Conclusion

**FIGURE 1:**
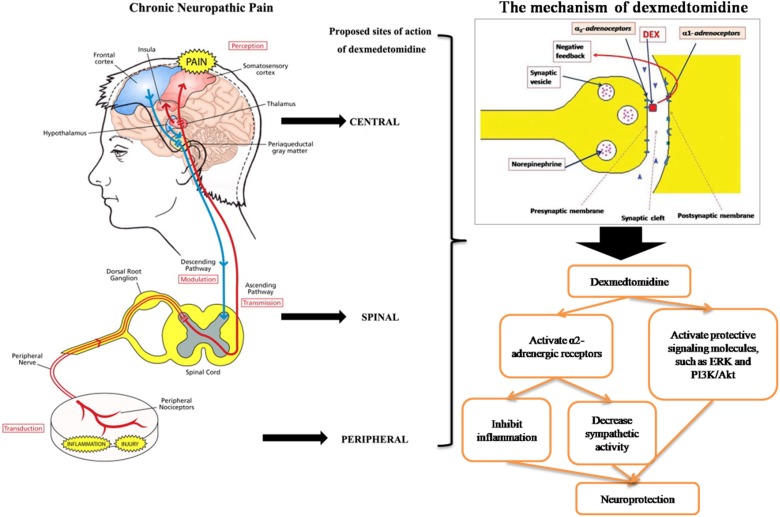
Dexmedetomidine for neuropathic pain.

Dexmedetomidine is a novel, highly selective α2 adrenergic receptor agonist with moderate analgesic effects (O’Neil et al., 2014). Both preoperatively and intraoperatively, dexmedetomidine can effectively alleviate the stress response caused by anesthesia, can maintain stable circulation, and can reduce the amount of anesthetic drugs (Greenberg et al., 2017). Dexmedetomidine has a good analgesic effect in animal models of acute and chronic inflammatory pain and postoperative pain and even in chronic pain patients for whom opioid analgesics are ineffective (Mantz et al., 2011). Although most studies have shown that dexmedetomidine is safe, dexmedetomidine may also have some complications, such as bradycardia (Zhang and Bai, 2014). Therefore, strict monitoring is required in clinical use. In this review, we summarized the neuroprotective mechanisms of dexmedetomidine (Figure 1), but studies on these mechanisms are mostly limited to animal experiments (Wang et al., 2019). Therefore, in future clinical research, it is necessary to consider the influence of various drug factors on various aspects of neuronal injury and to conduct a large number of preclinical experiments to accurately assess the safety and effectiveness of dexmedetomidine on the neuroprotective effects of the human body (Wang L. et al., 2017; Wang et al., 2019). Moreover, in lieu of the neuroprotective effect of dexmedetomidine, the drug has begun to be studied in clinical neuropathic pain diseases (Kim et al., 2017; Shi et al., 2018). Thus, we have also summarized many studies on the application of dexmedetomidine in neuropathic pain in this review.

## Author Contributions

YZ and SW designed the concept and drafted the manuscript. JH, NY, and CJ collected the literature, analyzed the data and edited the language in the manuscript.

## Conflict of Interest

The authors declare that the research was conducted in the absence of any commercial or financial relationships that could be construed as a potential conflict of interest.
